# Impaired IGF1-GH axis and new therapeutic options in Alström Syndrome patients: a case series

**DOI:** 10.1186/1757-1626-2-19

**Published:** 2009-01-07

**Authors:** Cristina Maria Mihai, Doina Catrinoiu, Marius Toringhibel, Ramona Mihaela Stoicescu, Negreanu-Pirjol Ticuta, Hancu Anca

**Affiliations:** 1 "Ovidius" University Constanta, Faculty of Medicine, Constanta County Emergency Hospital, 145 Tomis Blvd. 900591, Constanta, Romania; 2 "Ovidius" University of Constanta, Faculty of Pharmacy, 300, Mamaia Bvd., Constanta, Romania

## Abstract

**Background:**

Defects of the primary cilium and its anchoring structure, the basal body, cause a number of human genetic disorders, collectively termed ciliopathies: primary ciliary dyskinesia, Bardet-Biedl syndrome, polycystic kidney and liver disease, nephronophthisis, Alström syndrome, Meckel-Gruber syndrome and some forms of retinal degeneration.

Alström syndrome is an extremely rare, autosomal recessive genetic disorder characterized by a group of signs and symptoms including infantile onset dilated cardiomyopathy, blindness, hearing impairment/loss, obesity, diabetes, hepatic and renal dysfunction.

Because adult growth hormone deficiency and Alström Syndrome share some clinical and metabolic features, we studied the GH-IGF1 axis, using MRI techniques and dynamic tests in 3 unrelated patients with Alström syndrome.

**Case presentation:**

The patients were hospitalized and the growth hormone stimulatory tests were made, as well as brain MRI. Insulin provocative test revealed a severe GH deficiency in these patients, defined by a peak response to insulin-induced hypoglycemia less than 3 ng/dl and IGF1 concentrations less than – 2SDS.

We didn't find multiple pituitary hormone deficiency and we noticed only a severe GH deficiency in all three patients. The MRI study of the diencephalic and pituitary region was suggestive for the diagnosis of empty sella in one patient.

One patient received Recombinant-GH replacement for one year with very good results, one underwent a gastric sleeve with a satisfactory outcome, one patient died due to the progression of the cardiac myopathy.

**Conclusion:**

Future studies are needed to assses if the substitution therapy with Recombinant Growth hormone is cost-effective and without risk in such patients with Alström Syndrome and severe insulin resistance, despite our good results in one patient. Also, careful clinical and genetic studies can contribute to a better understanding of the evolution after different therapeutical attempt in the complex disorders such as Alström Syndrome.

## Background

Alström syndrome is a rare autosomal recessive disorder [1], caused by mutations in a gene of unknown function (ALMS1) [2] and it is characterized by several phenotypes reminiscent of Biedl-Bardet syndrome, including retinal degeneration, obesity and diabetes. ALMS1 protein localizes to centrosomes and to the base of cilia. In fibroblasts with disrupted ALMS1, primary cilia and the microtubule cytoskeleton appear to be normal, suggesting that the ALMS phenotype results from impaired ciliary function rather than from abnormal ciliary structure [2].

Central features of Alström syndrome include obesity, insulin resistance, and type 2 diabetes, and therefore investigating such patients could offer new insights into the pathogenesis of these common conditions [2].

Primary cilia are ubiquitous cellular appendages that provide important yet not well understood sensory and signaling functions. Until recently, cilia were thought to be simple external cellular organelles, but now they are thought to play important roles in cell signaling, in sensing chemical and physical activity, in intracellular communication, and as photoreceptors. The significance of primary cilia is exemplified by the fact that defects in cilia formation or function cause renal cystic disease, retinal degeneration, liver fibrosis, anosmia, ataxia, cardiac defects, and situs inversus [3,4].

Although all ciliopathies arise from defective cilia, the range of symptoms can vary significantly [5], and only a small subset of the possible ciliary disease symptoms may be present in any given syndrome, because the cilia are themselves exceedingly complex machines that perform multiple functions simultaneously [6-9].

There is growing evidence that cilia are connected with the cellular signaling involved in modern illnesses such as obesity and diabetes, e.g. an increasing number of genetic diseases being associated with defects in ciliogenesis or ciliary function [10-20], including Alstrom and Bardet-Biedl syndromes [2,18].

The complex links in the central nervous system between neuronal cilary dysfunction in the brain areas such as the hypothalamus, involved in appetite control, and obesity may be explained by the mechanisms of ciliary maintenance and lead to hyperphagia, a low metabolic rate, autonomic imbalance, growth hormone (GH) deficiency and various other problems that contribute to weight gain [21-24].

It is important to identify those at high risk of hypothalamic obesity so that weight gain prevention approaches can be offered. In those who are already obese, the principal causal mechanism should be considered as a basis for guiding clinical management [25-27].

Also, by studying such rare disorders we could understand the complex mechanisms of obesity and diabetes, were, dietary habits and exercise could be sometimes accompanied by genes linked to cilia and basal bodies, making some people more susceptible to obesity than others.

## Case presentation

Considering that hypothalamic ciliary neuronal dysfunction is implicated in the etiology of obesity in Alström syndrome patients, we studied the presence of GH deficiency in our patients, because we assume that an early preventive intervention in such patients is GH replacement.

We evaluated hypothalamic-pituitary-GH axis, by studying the GH-IGF1 axis, using MRI techniques and dynamic tests in 3 unrelated patients with Alstrom syndrome.

To characterize the GH-IGF1 system in Alström syndrome, we evaluated our 3 patients with Alström syndrome for hepatic, renal and thyroid function. Glycaemic and hormone measurements such as insulin, GH, FSH, LH, testosterone and 17-beta-oestradiol were assessed. A significantly lower height was observed in our patients compared to age-matched controls. Also, all patients were clinically obese (by weight and body mass index (BMI) standards). Only 2 patients presented with fasting hyperglycaemia, but all 3 were hyperinsulinaemic. TSH was normal. Baseline FSH and LH in the 2 females (Patient 2 and Patient 3) were within the normal range, while the male (Patient 1) had an abnormally low testosterone value. Free IGF1, IGFBP-1 were significantly reduced and IGFBP-2 was markedly increased in all 3 patients compared to age-matched controls, which points to a growth hormone deficiency (GHD) condition in Alstrom syndrome.

All 3 subjects had anthropometric and laboratory parameters checked at baseline. After a 12-hour overnight fast, blood specimens were obtained for plasma glucose, triglycerides, glycosylated hemoglobin (HbA1c), and serum total, low-density lipoprotein (LDL), and high-density lipoprotein (HDL) cholesterol. Patients who were using insulin (patient 1 and patient 2) had their treatment stopped the day before the test, but were closely monitored for the glycemic status.

An insulin tolerance test (ITT), consisting of a bolus of regular human insulin (0.1 U/kg body weight equivalent to 5.22 ± 0.44 U/m^2 ^of body surface) was performed in each patient.

At present, the insulin tolerance test is the diagnostic test of choice for the study of GH-IGF1 axis in obese patients. If provided adequate hypoglycemia is achieved, this test distinguishes GH deficiency from the reduced GH secretion that accompanies normal aging and obesity.

Most normal subjects respond to insulin-induced hypoglycemia with a peak GH concentration of more than 5 ng/dl. Severe GH deficiency is defined by a peak GH response to hypoglycemia of less than 3 ng/dl [28-30].

### Patient 1

**Figure 1:**
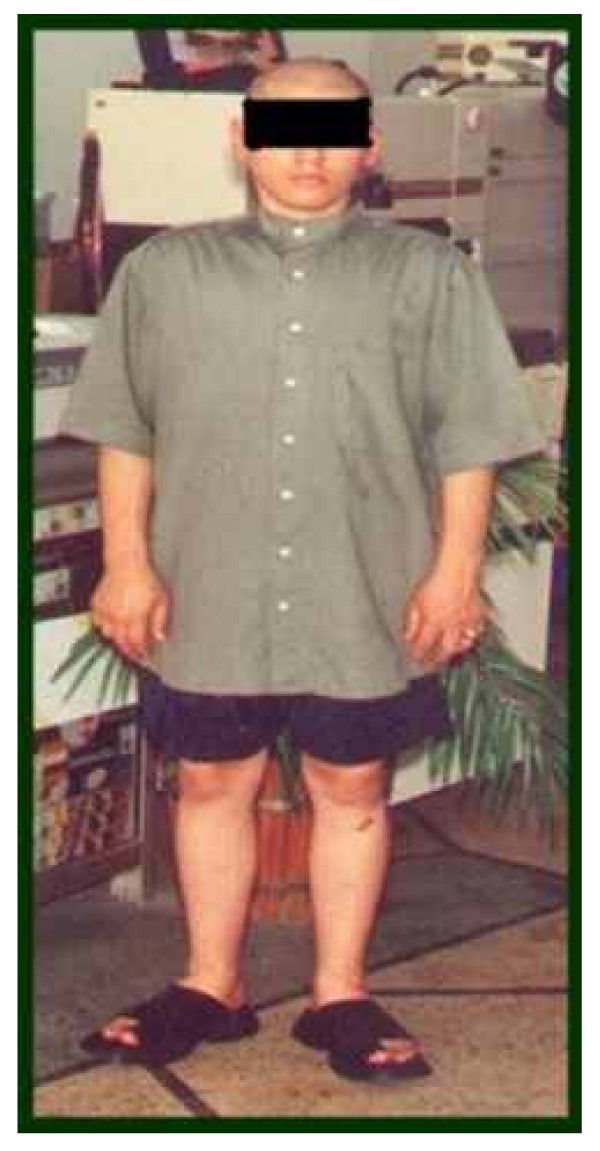
**Patient 1**.

The first patient diagnosed with Alström syndrome in our department was a 20-year-old boy, born from a non-consanguineous parents. During his first year of life were noted increased weight gain and progressive and worsening pathological ocular signs: nystagmus (at 4 months) and photophobia (at 12 months), reduced visual acuity. Sensorineural hearing impairment was noted at 15 years of age and type 2 diabetes mellitus was diagnosed at 16 years of age (Figure 1). From that point, he has been on insulin therapy, in different protocols, with 3 of 4 injections/day. The needed dose of insulin in order to obtain a good glycemic control was more than 200 IU/day. At this point, diabetes mellitus was considered as the result of resistance to the action of insulin. He never developed ketosis or diabetic keto-acidosis (DKA). Also, abnormal liver and kidney function tests were noted, an increased level of triglycerides with normal cholesetrol, with normal intelligence, normal extremities, normal secondary sexual characteristics. Systolic dysfunction of the left ventricle was diagnosed by echocardiography (long parasternal axis showed diffuse hypokinesia with mitral valve failure). Using Tissue Doppler Application, longitudinal velocity shortening and pulmonary hypertension 65–70 mmHg were also found.

**Figure 2:**
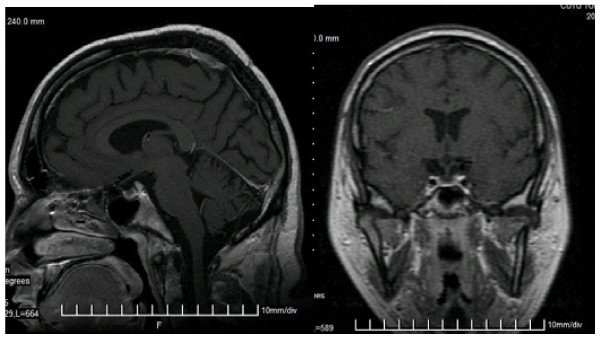
**Patient 1. **Magnetic resonance imaging – morphologic appearance suggesting “empty sella”.

We studied the GH-IGF1 axis, using MRI techniques and dynamic tests (insulin tolerance test). The MRI study of the diencephalic and pituitary region was suggestive for the diagnosis of empty sella (Figure 2). We found normal TSH (Thyroid Stimulating Hormone), fT3 (free triiodothyronine), fT4 (free thyroxine), and negative results for anti-thyroid auto-antibodies. Growth hormone stimulatory tests were made and insulin provocative test revealed a severe GH deficiency in this patient, defined by a peak response to insulin-induced hypoglycemia less than 3 ng/dl and IGF1 concentrations less than -2SDS.

The values were: GH1 – 0,18 ng/mL, GH_2 _– 0,49 ng/mL (after the stimulation with insulin 0,4 iu/kg/dose), IGF-1 – 49 ng/ml (N:116–356 ng/ml). Because of the insulin resistance, the needed dose of insulin in order to achive the hypoglycemia, was 4 times greater than usual.

Multiple pituitary hormone deficiency (MPHD) wasn't found and we noticed only severe GH deficiency and an abnormally low testosterone value. The presence of empty sella seems to be a rare morphologic finding in Alström syndrome though it was described in patients with Bardet-Biedl syndrome [31].

Decreased serum levels of GH, which acts on cardiac myocytes primarily through IGF-1, are associated with impaired myocardial growth and function, which can be improved with restoration of GH/IGF-1 homeostasis [32]. This effect of GH on cardiac tissue may explain why the patient had multiple episodes of cardiac decompensation due to severe cardiomyopathy and finally died at 28 years of age.

Also, due to the severity of the cadiomyopathy, we were reluctant to consider the Re-GH administration in this case.

The patient was included in a study of linkage mapping (Jackson Laboratory, Bar Harbor, Maine). Interestingly, so far, the Alström patient doesn't have any of the known ALMS1 mutations and he did not have any of the known BBS mutations either. He had one heterozygous SNP in BBS5 (BBS5_N184S het) that is not disease-causing. Despite the fact that this case was analyzed for years without the identification of a known mutation, efforts are still made using the Asper Biotech Gene Chip, in order to identify a possible new unknown mutation(s) that may explain the phenotypic diagnosis.

### Patient 2

The second patient is a girl, 14-y-o, diagnosed with Alström syndrome clinically and genetically (Jackson Laboratory, Bar Harbor, Maine, USA). The genetic testing performed at Jackson Laboratory, Bar Harbor, Maine revealed in exon 16, in the ALMS1 gene in patient's DNA, 2 different mutations: 10753 C > T Ter and 10780 C > T Ter.

The first sign, nystagmus, was noted at 4 months of age, then she developed progressive retinal distrophy and blindness by 10 years of age. Infantile obesity progressive with age, sensorineural hearing impairment, type 2 diabetes mellitus, abnormal liver function tests, reccurent urinary tract infections, urinary incontinence, scoliosis, kyphosis, acanthosis nigricans, obesity (123 kg/167 cm, BMI = 44.1 kg/m^2^) but, normal intelligence, normal extremities, normal secondary sexual characteristics were noted during the evolution.

We studied the GH-IGF1 axis, using MRI techniques and dynamic tests (insulin tolerance test).

We found a severe GH deficiency, defined by a peak response to insulin-induced hypoglycemia less than 3 ng/dL and IGF1 concentrations less than -2SDS.

The values were: GH1 – 0,18 ng/mL, GH2 – 0,17 ng/mL (after stimulation with insulin 0,2 IU/kg/dose), IGF-1 (somatomedin c) – 67 ng/mL (116–356).

Because of the insulin resistance, the needed dose of insulin in order to achive the hypoglycemia, was 2 times greater than usual.

**Figure 3:**
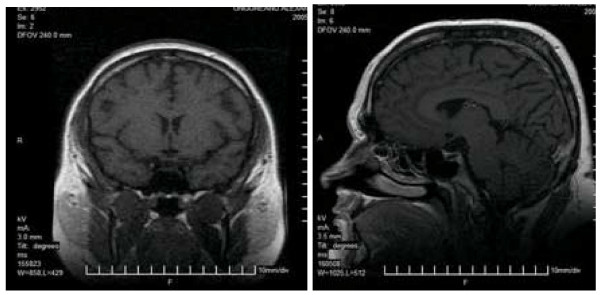
**Patient 2**. Normal MRI study of the diencephalic and pituitary region.

The MRI study of the diencephalic and pituitary region was normal (Figure 3).

We found normal TSH (Thyroid Stimulating Hormone), fT3 (free triiodothyronine), fT4 (free thyroxine), and negative results for anti-thyroid auto-antibodies. Multiple pituitary hormone deficiency (MPHD) wasn't found and we noticed only severe GH deficiency.

**Figure 4:**
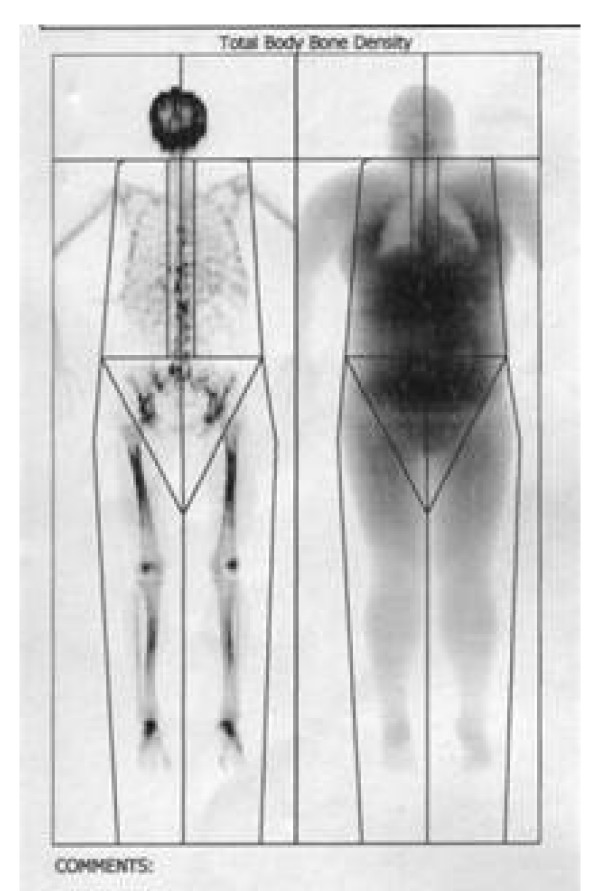
**DEXA body composition analysis**. Patient 2. Height/Weight: 167 cm/120 kg. Sex/Etnic: Female/White. Total region = 1,158 g/cm^2 ^BMD1

**Figure 5:**
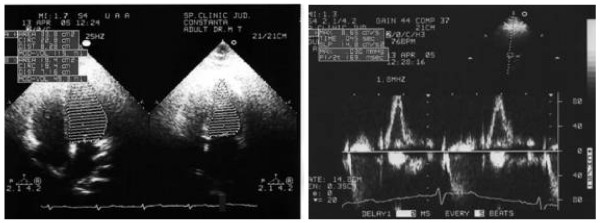
**Patient 2**. Echocardiography: left ventricular mass index, fractional shortening and fiber shortening velocity improved after 12 month of low-dose therapy with Re-GH.

We used Re-GH in this patient for 12 consecutive months, in a dose of 0,25 mg/day, with very good results: the total body fat mass decreased after one year, most significant in visceral trunk location as revealed by DEXA body composition, the bone density increased with 5% after 6 months (Figure 4). Echocardiography has shown that left ventricular mass index, fractional shortening and fiber shortening velocity improved after 12 month of low-dose therapy (Figure 5). No lipid metabolism improvement was noted, but a psychological well being was observed. Insulin sensitivity and acanthosis nigricans improved. Glucose-to-insulin ratio increased from 3.45 to 4.52 after one year.

### Patient 3

**Figure 6:**
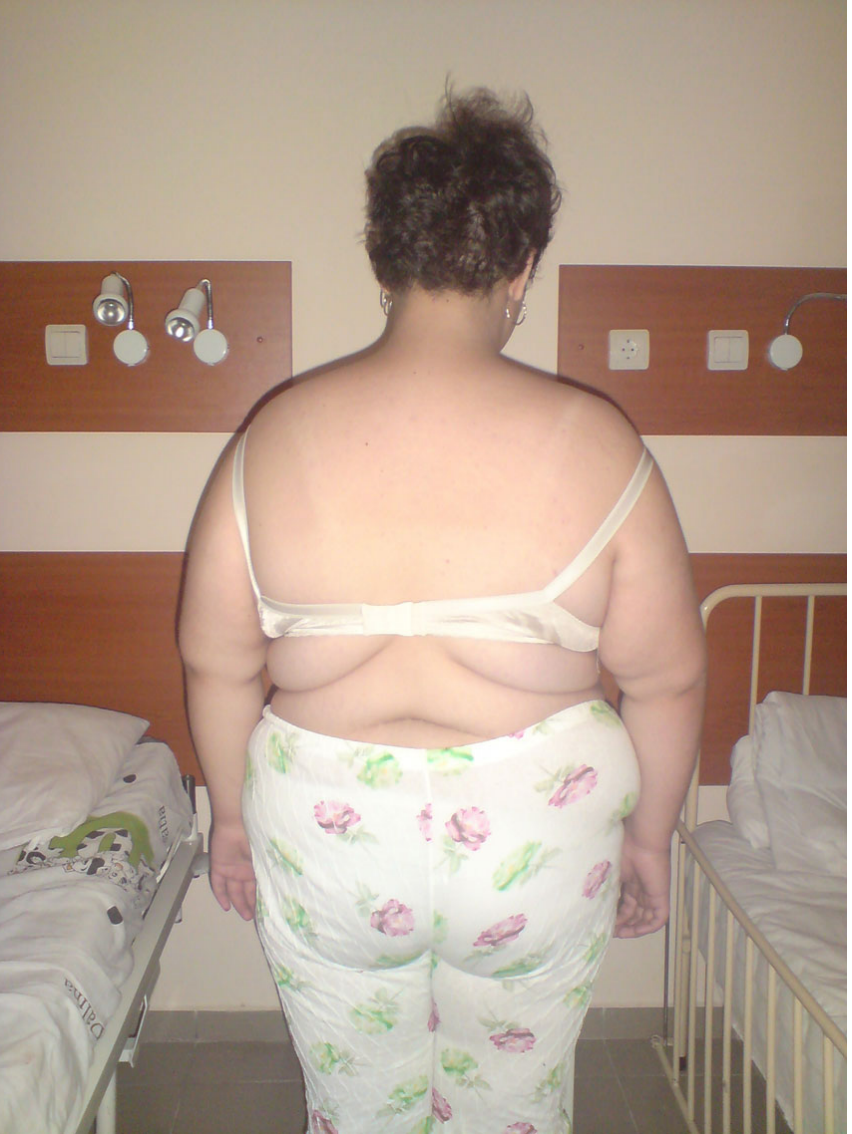
**Patient 3**.

The third patient is a 13-y-o girl with severe obesity (Figure 6) who was examined in our department after the gastric sleeve was performed for her severe obesity resistant to diet and medical treatment for years.

The past medical history revealed the presence of nystagmus from 4 months of age and progressive obesity during her infancy and childhood. The patient has normal extremities and normal inteligence, no sensorineural hearing impairment was noted yet, nor type 2 diabetes mellitus, and thyroid disorders. Differential diagnostics included lysosomal storage diseases and aminoacidopathies, but these disorders were excluded. Several laboratory tests showed normo-glycemia, mild hypertriglyceridemia, and hypercholesterolemia and normal liver transaminases and renal function tests. Mild to moderate insulin-resistance was noted, as revealed by glucose-to-insulin ratio (GIR) = 4.3 Obese patients with a fasting GIR of 4.5 or more have insulin sensitivity, while a GIR of less than 4.5 means insulin resistantance. Fasting Insulinemia: 18.5 uU/mL (Normal: 2.6–24.9). We studied the GH-IGF1 axis, using MRI techniques and dynamic tests (insulin tolerance test): GH 1- 0.32 ng/mL, GH 2- 0.24 ng/mL (when hpoglycemia was induced, glycemia < 40 mg/dl), IGF-1 162 ng/mL (Normal: 220–972).

Insulin resistance as well as growth hormone deficiency was diagnosed in this patient; her DNA was sent to Jackson Laboratory, Maine, USA for the genetic testing and we are pending for the results.

Despite the gastric sleeve and considerable weight loss (about 45 kg in 9 months), this patient was diagnosed with GH deficiency unrelated to obesity, and we are considering the Re-GH administration in her case.

We noticed an important clinical variability among these patients, characteristic for the ciliopathies, given the multiple roles of cilia in development and physiology.

The first sign noticed in all these 3 affected patients was the presence of nystagmus and light sensitivity in infancy.

Congestive heart failure resulting from dilated cardiomyopathy was diagnosed in patient 1 in adolescence, leading to his death, by the age of 28. Also, the cardiomyopathy was diagnosed in patient 2, but some improvements were noted after the treatment with Re-GH.

As infants and toddlers all 3 patients were overweight. Hearing impairment was diagnosed in patients 1 and 2, and all 3 became insulin resistant as adolescents. Patient 1 and patient 2 were diagnosed with type 2 diabetes mellitus in the 2-nd decade of life.

Other findings observed in our Alström Syndrome patients: acanthosis nigricans, short stature, abnormal liver and kidney tests in patients 1 and 2, and normal intelligence, normal extremities, normal secondary sexual characteristics in all 3 patients, making possible the differential diagnosis with Bardet-Biedl syndrome.

Patient 1 was diagnosed with empty sella, based on the brain MRI exam, this finding being unusual so far in Alström Syndrome patients.

Studying the medical literature, we noticed that considerable variability exists in the expression of the clinical signs of Alström Syndrome, even among siblings, and not all people who have Alström Syndrome will experience all of the characteristic features [27].

All 3 patients in our series were diagnosed with GH deficiency, based on the results of the provocative tests using insulin, one problem of great concern being the obesity.

The somatotropic response to all provocative stimuli is negatively correlated to BMI and the GH response in obese subjects is sometimes as impaired as that in hypopituitary patients with severe GHD [33-35]. The impairment of GH secretion in obese would reflect alterations in the neuroendocrine control of the somatotropic axis and/or metabolic alterations such as hyperinsulinism and elevation of circulating free fatty acids [36]; reduction of GH half life in obese subjects has also been demonstrated [37,38]. For the ITT, the classical cut off level of 3 ng/dl can be considered appropriate based on well known evidence that this level distinguishes normal subjects (including obese) from patients with severe GHD [33,34], in order to avoid false positive diagnosis in obese adults.

We used the ITT and the classical cut off level of 3 ng/dl when we assessed the impairment of GH secretion in our patients.

It is supposed that the genetic alteration leads to dysfunction of several hypothalamic centres and growth hormone (GH) deficiency (GHD) in Alström syndrome.

In our case series, at baseline the activity of the GH-insulin-like growth factor-1 (IGF-1) system was impaired with low GH values, low total IGF-1 and in relation to the obesity low levels of free IGF-1 and non-suppressed IGF-binding-protein-1 (IGFBP-1). Bone mineral density (BMD) assesed in one patient was low. Two patients had diabetes mellitus, and all three had a glucose-to-insulin ratio (GIR) less than 4.5 indicating insulin resistance. One patient subsequently completed a 12 months GH treatment trial, and GH had beneficial effects on body composition without significant adverse effects. More, the echocardiography performed in this patient has shown that left ventricular mass index, fractional shortening and fiber shortening velocity improved after 12 month of low-dose therapy. No lipid metabolism improvement was noted, but a psychological well being was observed. Insulin sensitivity and acanthosis nigricans improved. Glucose-to-insulin ratio increased from 3.45 to 4.52 after one year.

Demonstrating GHD in an Alström syndrome patient, Tai TS, Lin SY, Sheu WH assessed the metabolic effects of growth hormone therapy concluding that Re-GH therapy might have beneficial effects on body composition, liver fat content, lipid profiles, and insulin resistance in Alström syndrome patients, with improvement of the glucose homeostasis [39].

Also, Maffei et al. found a reduction of ALS (acid labile subunit) and the increase of IGFBP-2 as expression of growth hormone deficiency (GHD) condition in 15 young adults with Alström syndrome [40].

Considering Recombinant-GH as treatment in to 8 pediatric patients with stable chronic heart failure secondary to dilated cardiomyopathy (DCM), McElhinney and the other authors determined several notable cardiovascular effects, including a trend toward improved LV ejection fraction during the course of GH treatment and significantly improved LV SF, SF *z *score, and LV end systolic stress *z *score 6 months after discontinuation of GH treatment (relative to baseline values) [41]. Re-GH wasn't tried so far in Alström syndrome patients with cardiac myopathy, as an alternative to the classical treatment.

Concerning the gastric sleeve, we consider that this procedure should be addressed to extremely obese patients with Alstrom syndrome, (typically with BMI greater than 40), who have reached their adult height (usually 13 or older for girls and 15 or older for boys), and have serious weight-related health problems, such as type 2 diabetes, sleep apnea, heart disease, or significant functional or psychosocial impairment. In addition, potential patients and their parents should be evaluated to see how emotionally prepared they are for the operation and the lifestyle changes they will need to make.

## Conclusion

This case series showed that adolescents and adults with Alström syndrome have GH deficiency, and recombinant GH therapy might have beneficial effects on body composition, and insulin resistance, with improvement of the glucose homeostasis and cardiac function. Larger and longer term studies on the effect of GH replacement in Alström syndrome patients are encouraged, to assess if the substitution therapy with Recombinant Growth hormone is cost-effective and without risk in such patients with Alström Syndrome and severe insulin resistance, knowing that the state of GH deficiency seen in Alström syndrome renders these patients exposed to a lifelong risk of metabolic disturbances.

Further careful clinical and genetic studies of such patients can contribute to a better understanding of the evolution after different therapeutical attempts in the complex disorders such as Alström Syndrome.

## Abbreviations

GH: growth hormone; IGF-1: insulin-like growth hormone 1; GHD: growth hormone deficiency; MRI: magnetic resonance imaging; SDS: standard deviation; MPHD: multiple pituitary hormone deficiency; ALMS1-Alström syndrome 1(the gene responsible for Alström Syndrome); DKA: diabetic keto-acidosis; TSH: Thyroid Stimulating Hormone; fT3: free triiodothyronine; fT4: free thyroxine; DEXA: Dual energy X-ray absorptiometry; BMI: body mass index; BMD: bone mineral density; ITT: insulin tolerance test; SNP: single nucleotide polymorphism; BBS5: Bardet-Biedl Syndrome 5 (a human gene); GIR: glucose-to-insulin ratio; Re-GH: recombinant growth hormone; ALS: acid labile subunit; DCM-dilated cardiomyopathy.

## Consent

Written informed consent was obtained from the patients's parents for publication of this article and accompanying images. A copy of the written consent is available for review by the Editor-in-Chief of this journal.

## Competing interests

The authors declare that they have no competing interests.

## Authors' contributions

RMS and HA were responsible for analyses of blood samples and initial drafting of the manuscript. TNP was responsible for design, planning, execution and drafting of the manuscript. CMM and DC were involved in drafting the manuscript and revising it critically for intellectual content. MT was responsible for the cardiac echography.
